# Canary in the Coal Mine: How Resistance Surveillance in Commensals Could Help Curb the Spread of AMR in Pathogenic *Neisseria*

**DOI:** 10.1128/mbio.01991-22

**Published:** 2022-09-26

**Authors:** Maira Goytia, Crista B. Wadsworth

**Affiliations:** a Department of Biology, Spelman College, Atlanta, Georgia, USA; b Inserm, System Engineering and Evolution Dynamics, Université Paris Cité, Paris, France; c Learning Planet Institute, Paris, France; d Rochester Institute of Technology, Thomas H. Gosnell School of Life Sciences, Rochester, New York, USA; Ohio University; Ohio State University

**Keywords:** *Neisseria*, commensal bacteria, horizontal gene transfer, antibiotic resistance, microbiome, *N. gonorrhoeae*, *Neisseria gonorrhoeae*

## Abstract

Antimicrobial resistance (AMR) is widespread within Neisseria gonorrhoeae populations. Recent work has highlighted the importance of commensal *Neisseria* (cN) as a source of AMR for their pathogenic relatives through horizontal gene transfer (HGT) of AMR alleles, such as mosaic *penicillin binding protein 2* (*penA*), *multiple transferable efflux pump* (*mtr*), and *DNA gyrase subunit A* (*gyrA*) which impact beta-lactam, azithromycin, and ciprofloxacin susceptibility, respectively. However, nonpathogenic commensal species are rarely characterized. Here, we propose that surveillance of the universally carried commensal *Neisseria* may play the role of the “canary in the coal mine,” and reveal circulating known and novel antimicrobial resistance determinants transferable to pathogenic *Neisseria.* We summarize the current understanding of commensal *Neisseria* as an AMR reservoir, and call to increase research on commensal *Neisseria* species, through expanding established gonococcal surveillance programs to include the collection, isolation, antimicrobial resistance phenotyping, and whole-genome sequencing (WGS) of commensal isolates. This will help combat AMR in the pathogenic *Neisseria* by: (i) determining the contemporary AMR profile of commensal *Neisseria*, (ii) correlating AMR phenotypes with known and novel genetic determinants, (iii) qualifying and quantifying horizontal gene transfer (HGT) for AMR determinants, and (iv) expanding commensal *Neisseria* genomic databases, perhaps leading to the identification of new drug and vaccine targets. The proposed modification to established *Neisseria* collection protocols could transform our ability to address AMR N. gonorrhoeae, while requiring minor modifications to current surveillance practices.

## INTRODUCTION

Antimicrobial resistance (AMR) in Neisseria gonorrhoeae is an urgent threat to public health, with the emergence of resistance in gonococcal populations to all antimicrobials that have been recommended for treatment (1), and infection rates simultaneously on the rise (2). According to the World Health Organization (WHO), an estimated 86.9 million people were infected by the gonococcus worldwide in 2016 (3). In the most recent United States survey by the Gonococcal Isolate Surveillance Project (GISP) from the U.S. Centers for Disease Control and Prevention (CDC), only 44.5% of isolates remained susceptible to all tested antimicrobials (2). Furthermore, treatment failures to dual antimicrobial therapy (ceftriaxone plus azithromycin) have been reported internationally (4–6). This, coupled with a precipitous rise in azithromycin resistance (7), has prompted a change in recommended treatment regimen for uncomplicated gonococcal infection, from dual therapy with ceftriaxone (single intramuscular injection of 250 to 500 mg) and azithromycin (single oral dose of 1 to 2 g), to ceftriaxone monotherapy (single 500 mg intramuscular dose) (8). With only two novel therapeutics currently in development (i.e., zoliflodacin [9] and gepotidacin [10]), new strategies must be implemented to combat the growing threat of AMR in N. gonorrhoeae.

Worldwide, several national and international surveillance programs, such as GISP from the CDC, and the global Gonococcal Antimicrobial Surveillance Program (GASP) and its European version (Euro-GASP) from the WHO, aim to control and prevent the spread of AMR in N. gonorrhoeae. The identification of resistance mechanisms to first-line antibiotics circulating within gonococcal populations has been emphasized in recent years (11–16); however, these efforts often overlook a known source of resistance for gonococci—the commensal *Neisseria* (4–7). In addition to the two human pathogens within the *Neisseria* genus (N. gonorrhoeae and N. meningitidis), there are, at minimum, eight closely related commensal *Neisseria* species carried harmlessly in healthy human adults and children (17–19) that rarely cause disease (20). To note, N. meningitidis is considered an obligate human commensal and opportunistic pathogen, as it is commonly carried in the population asymptomatically but can cause invasive life-threatening disease. Commensal species share alleles with their pathogenic relatives, and genetic mosaicism has been observed genome-wide, whereby loci containing full or partial genes or regulatory sequences within a particular lineage have been acquired from another *Neisseria* species (4–6, 13, 15, 16, 21, 22). Natural competence, the ability of *Neisseria* to uptake and integrate DNA at any point during growth and without a necessary external stimulus, as well as high rates of recombination has led many to question the nature of species’ delineations within the genus (11, 23). Likewise, promiscuous allelic exchange has repeatedly been documented to have facilitated rapid adaptive evolution of important phenotypic characteristics such as antimicrobial resistance (4, 21, 24, 25) and body-site colonization niche shifts (16). Thus, without detailed characterization of the resistance alleles in commensals and assessment of their population prevalence, we are blinded to novel mechanisms of resistance in commensal *Neisseria* that may rapidly and unknowingly disseminate to the pathogenic *Neisseria.*

A current challenge to inclusion of commensals in genus-level population resistance monitoring is the relative undersampling of commensal populations compared with their pathogenic relatives (20, 26). The diversity of AMR alleles in commensal populations is currently unknown, presenting an issue for complete genotypic-based resistance prediction for any of the *Neisseria* species, including pathogens. Improved characterization of the *Neisseria* resistome (i.e., total collection of all resistance alleles available to members of the genus), and subsequent surveillance of commensal populations could serve as a “canary in the coal mine” approach to identifying new or common resistance mechanisms that may emerge from our natural microbial reservoirs. Kenyon et al. have previously championed a call for surveillance of commensal *Neisseria* as part of their model considering a “pan-*Neisseria*” approach for choosing appropriate antibiotic therapy for gonorrhea (27), which we expand upon here as an important next step in addressing AMR within the pathogenic *Neisseria.* Collection and characterization of commensal *Neisseria* isolates as part of routine surveillance programs by clinical and academic laboratories should provide broad epidemiological surveillance of AMR in commensals, as the carriage rate of commensals (around 100%) is higher than that of their pathogenic relatives (0.01% to 10%) (28–30), and we believe could ultimately serve as an early warning indicator of possible resistance outbreaks in pathogenic *Neisseria.*

Here, we review the background on commensal *Neisseria*, horizontal gene transfer within the genus, and the clinically relevant resistance alleles in the pathogenic *Neisseria* that have been acquired from commensal species. Furthermore, we summarize the current literature on commensal resistance, both the known mechanisms and published surveillance efforts. Finally, we highlight the necessary steps that must be taken to successfully enhance our ability to detect and predict resistance in and from nonpathogenic reservoirs.

## THE *NEISSERIA* GENUS: OUR BIAS TOWARD THE HUMAN PATHOGENS

The genus *Neisseria* is comprised of several closely related Gram-negative species, which are typically diplococcoid, oxidase-positive and often catalase-positive, and usually isolated from mucosal membranes of humans and animals (20) (only human-associated *Neisseria* will be reviewed here). Phylogenomic analyses suggest that all human-associated *Neisseria* evolved from a common ancestor which colonized the oral cavity of an early humanoid (31, 32). Interestingly, metagenomic surveys have demonstrated clear specificity of contemporary *Neisseria* species to distinct sites within the oro- and nasopharynx, also correlated to clades with different DNA uptake sequence motifs (DUS; see below for further discussion), suggesting distinct ecological niches have resulted in subsequent speciation and genetic divergence (33). Though there are eight broadly cited human-associated commensal species (Neisseria cinerea, N. polysaccharea, N. lactamica, *N. mucosa*, *N. oralis*, *N. subflava*, *N. elongata* [atypical rod], and *N. bacilliformis* [atypical rod]), three distinct genetic clusters within the N. polysaccharea species group and seven novel *Neisseria* species with distinct biochemical and genomic characteristics have recently been identified through phylogenomic analyses (26), suggesting that human commensal *Neisseria* diversity is likely greater than we currently know and could very likely surpass 20 species (26). Commensal *Neisseria* are considered part of the “core” oropharyngeal flora, with metagenomic surveys typically identifying *Neisseria* as the most abundant genus within proteobacteria (~10% of operational taxonomic units [OTUs]) in oral and pharyngeal samples (34, 35). Although correlations between abundance of *Neisseria* commensals and disease, and systemic infections have been reported, the commensal *Neisseria* are generally considered nonpathogenic members of the human microbiome (20, 36, 37).

Of the human-associated *Neisseria*, only N. gonorrhoeae (the gonococcus) and N. meningitidis (the meningococcus) cause disease at notable frequencies. N. meningitidis is an obligate human commensal that typically colonizes the nasopharynx and is carried asymptomatically in 10% of adults; however, it can occasionally become an opportunistic pathogen causing invasive meningococcal disease (meningococcemia) and bacterial meningitis (38, 39). N. gonorrhoeae is the only species within the genus that, in addition to the nasopharyngeal mucosa, also routinely colonizes the urogenital tract and rectum, and causes the sexually transmitted infection gonorrhea (40). N. gonorrhoeae and N. meningitidis are the closest phylogenetic relatives within the genus; and occasional reports of meningococcal urogenital colonizing strains (16, 41) has led to speculation that the two pathogenic *Neisseria* arose from a common ancestor (likely inhabiting the pharynx), which established in the urogenital niche and developed into the modern gonococcal lineage through adaptive evolution of traits such as the induction of the polymorphonuclear leukocyte (PMN) inflammation response aiding in sexual transmission (42), and nitrite-dependent anaerobic growth which may aid in urethral colonization (16, 43).

Research on commensal *Neisseria* is in its infancy compared with the pathogenic *Neisseria* species, N. gonorrhoeae and N. meningitidis. A PubMed query as of June 2022 revealed 25,902 articles in English including the search term “Neisseria” in the title, 12,859 articles including the search terms “Neisseria AND gonorrhoeae NOT commens*” compared with 334 articles with the search terms “Neisseria AND commens* NOT gonorrhoeae,” 11,036 articles including the search terms “Neisseria AND meningitidis NOT commens*,” 230 articles including the search terms “Neisseria AND commens* NOT meningitidis,” and 141 articles with the search terms “Neisseria AND commens* NOT gonorrhoeae NOT meningitidis.” Though restricted in scope, this analysis of the available literature captures the limited research focus, sampling, and sequencing (as pointed later in the text), on the commensals which emphasizes a weakness in our understanding of *Neisseria* AMR mechanisms that may be, as of yet, undiscovered with the full diversity of the genus undocumented.

## DNA PROMISCUITY: THE PREVALENCE OF ALLELIC EXCHANGE

Bacteria replicate asexually through binary fission, in which the chromosome(s) duplicate and subsequently segregate into unique daughter cells. This produces clonal population structures, with relatively low genetic diversity outside the variation created through the accumulation of beneficial or neutral *de novo* mutations over time (23, 44). This typical clonal structure, however, is disrupted by species, like *Neisseria spp*, that engage in horizontal gene transfer (HGT) through natural competence. Species capable of natural competence uptake naked extracellular DNA using specialized machinery and integrate homologous tracts into their genomes via RecA-mediated recombination (24), which reassorts alleles across lineages and allows adaptive genetic variation to rapidly spread within and between species (23, 44).

*Neisseria* are one of the most recombinogenic bacterial genera, with extensive allele sharing observed within and between species in the genus, breaking clonality and resulting in panmictic population structures (23, 44). In part, this is due to the fact that *Neisseria* are naturally competent for transformation through all stages of growth (45), and constitutively express their competence systems as opposed to many other recombinogenic bacteria like Bacillus subtilis (46), Haemophilus influenzae (47), or Streptococcus pneumoniae (48). The frequency of intragenus genetic exchange has led to the concept of *Neisseria* as an interconnected consortium of species with “fuzzy” borders (14, 23, 44, 49). Allelic exchange via this mechanism has introduced novel DNA tracts, estimated at 6% genome-wide, into gonococci from other members of the *Neisseria* genus (14, 22, 50, 51). Examples of intragenus allele sharing have been documented between different species pairs at the loci encoding the IgA proteases (21), Penicillin Binding Protein 2 (encoded by *penA*, discussed below) (5, 52), the Multiple Transferable Efflux Pump (*mtr*, discussed below) (13, 15), ribosomal protein genes (discussed below) (51, 53, 54), and *ornithine carbamoyltransferase* (*argF*) (6) among others.

Genetic exchange across species’ boundaries has facilitated rapid adaptive evolution of important phenotypic characteristics such as resistance to antibiotics (4, 21, 24, 25) and macrohabitat niche shifts (16). For example, in 2015 in the United States, several cases of men with urethritis caused by Gram-negative diplococci were nucleic acid amplification test (NAAT) negative for N. gonorrhoeae in Ohio and Michigan (16, 41). Further amplification tests revealed the cause to be a clonal group of serogroup C N. meningitidis strains which had acquired the gonococcal *aniA*/*norB* cassette. A nitrite reductase (AniA) and a nitric oxide reductase (NorB) are important components of an effective denitrification pathway and are essential for the anaerobic growth typical to the urogenital tract (55–57). N. gonorrhoeae isolates have functional *aniA* and *norB* loci, whereas the majority of meningococci which typically experience aerobic conditions in the nasopharynx, have frameshift mutations in *ani*A and cannot grow anaerobically using nitrite as the electron acceptor (58). However, introduction of a functional in-frame *aniA*/*norB* cassette from N. gonorrhoeae has directly led to adaptation to anaerobic conditions and the expansion of urogenital-colonizing N. meningitidis (59), speaking to the incredible power of HGT in facilitating large and rapid evolutionary shifts. Overall, recently incorporated mosaic sequences across *Neisseria* genomes show signatures consistent with positive selection (e.g., linkage of nearby nonsynonymous mutations, increased intermediate-frequency allele diversity, etc.), suggesting recombination is an important source of beneficial genetic variation through the introgression of coadapted allelic complexes on coinherited DNA tracts from close relatives (14).

The likely site for the bulk of cross-species gene exchange is in the oro- and nasopharynx, where most *Neisseria* species typically reside in proximal yet distinct ecological niches (33). Here, exogenous DNA uptake is mediated by functional type IV pili (Tfp). Tfp expression greatly enhances transformation efficiency in N. gonorrhoeae, with piliated strains exhibiting transformation frequencies in excess of 20% in the presence of high DNA concentrations (60), whereas piliation loss decreases these frequencies closer to 1 × 10^−7^ (61). Pili are embedded in the outer membrane and protrude into the extracellular milieu through the multimeric PilQ pore complex (62, 63), and are formed by pilin subunits (PilE and ComP) which assemble into a helical structure with a conserved core, central layer, and hypervariable outer region (64–66). Competence is facilitated by a protein exposed on the surface of the pilus, ComP, which has high affinity for *Neisseria*-specific DNA uptake sequences (DUS) (67–69). These conserved clade-specific 12-bp sequences (e.g., AT-DUS: 5′-AT-GCCGTCTGAA-3′), located every ~1,100 bp throughout *Neisseria* genomes (70), greatly increase transformation efficiency in *Neisseria* species with the same sequence motif “dialects.” The cytoplasmic NTPase motors PilF (also known as PilB), PilT, and PilU are involved in regulating pilus assembly and retraction (71–73). Retraction of pili is necessary for competence and is thought to facilitate transport of ComP-bound DNA through the outer membrane and peptidoglycan layer (74). Single-stranded DNA is then imported through the inner membrane and into the cytoplasm through a pore formed by ComA (75). Finally, two RecA-mediated pathways (RecBCD and RecF) integrate the DNA into the chromosome through homologous recombination (76–78).

Although interspecies recombination can provide rapid access to new advantageous alleles, it can also introduce variants that are deleterious in divergent genomic backgrounds through gene disruption or the creation of maladapted gene combinations (i.e., locus A + locus B = advantageous combination; locus C + locus D = advantageous combination; however, locus A + locus D = deleterious combination). Thus, there are some barriers to allelic exchange that do exist within the genus that limit transfer from more evolutionarily distant relatives. For example, coevolution of DUS motifs and their binding protein ComP (79) has contributed to the emergence of “reproductive isolation” between clades of *Neisseria* species. Eight sequence variations of DUSs coupled with alternative forms of the ComP protein create divergent “dialects,” which directly increase the frequency of DNA binding, uptake, and transformation within groups compared with between groups (70). A second barrier to recombination is divergence in the number and identity of methyltransferases between pathogenic and commensal *Neisseria* species. Compared with N. gonorrhoeae which harbors 14 to 19 DNA methyltransferases (80–82), the commensal *N. elongata* encodes only 7 to 10. Divergence in methyltransferase activity across these species differentially modifies CpG and GpC sequence motifs producing species-specific methylation signatures. Mismatched methylation has been demonstrated to kill *Neisseria* which have recently integrated novel DNA into their chromosomes through restriction enzyme(s) cleavage and the formation of double-strand breaks (83, 84). However, despite the aforementioned barriers, observable genome-wide recombination (14, 22, 85) and reports of specific alleles acquired across species’ borders (86, 87) directly support the importance of commensals as reservoirs of readily available adaptive genetic variation for the pathogens within the genus.

## COMMENSALS AS A KEY RESERVOIR OF RESISTANCE

**FIG 1:**
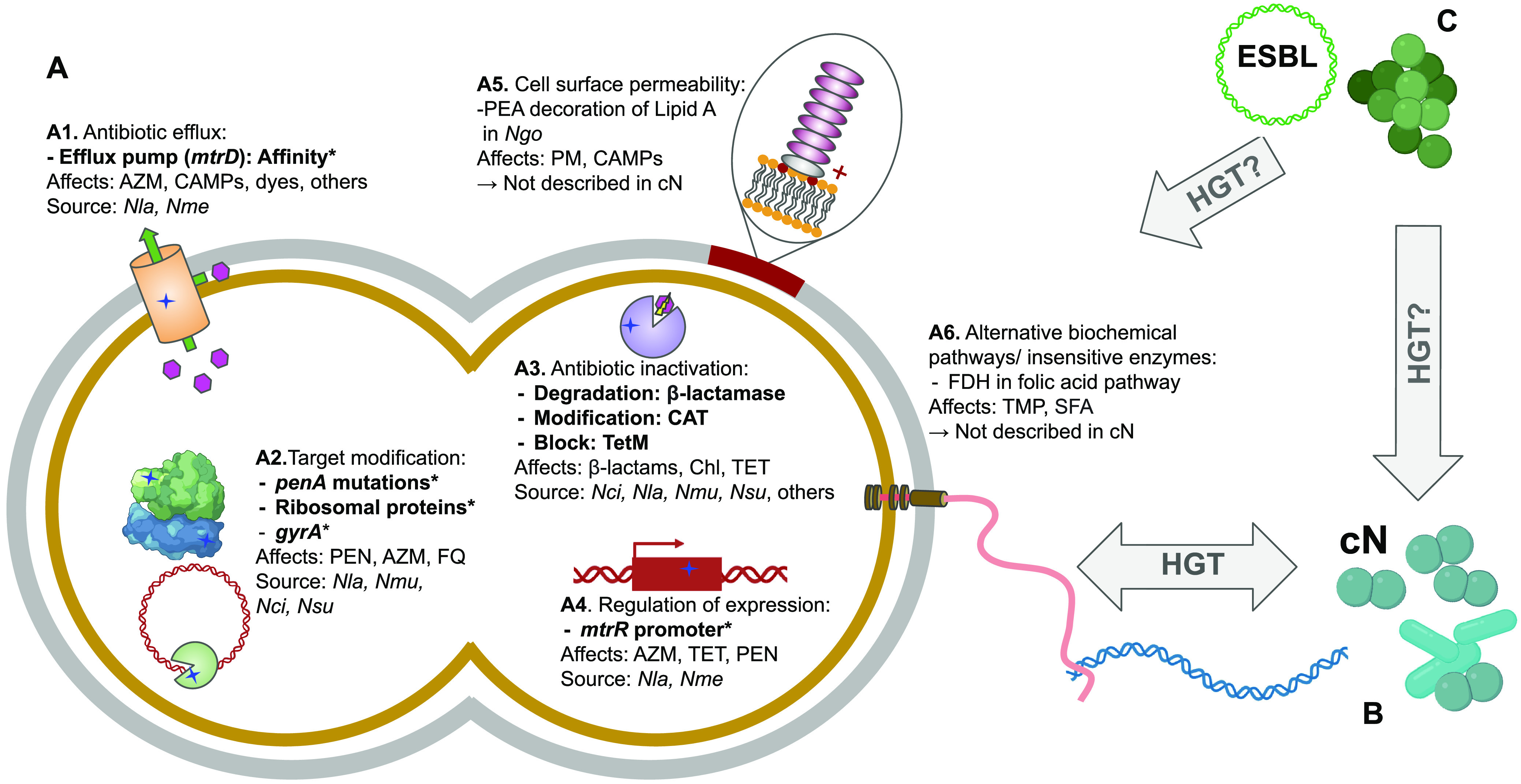
(A) Schematic representation of some of the known mechanisms of resistance in N. gonorrhoeae, including those that have been acquired from (bold text and *) or have been documented to be present in the commensal *Neisseria* (bold text). Known resistance encompasses multiple mechanisms, including: (A1) mutations enhancing the affinity of antibiotics for the Mtr efflux pump, increasing their efflux from the cell; (A2) mutations altering the structures of the targets of antibiotics reducing binding affinity (e.g., β-lactams [PEN] and PBP2, azithromycin [AZM] and ribosomal proteins, and fluoroquinolones [FQ] and DNA Gyrase Subunit A); (A3) genes encoding enzymes that modify antibiotics through degradation or blocking their target site; (A4) altered promoter motifs which increase or decrease the expression of genes; (A5) changes in cell surface permeability through mechanisms such as the decoration of Lipid A with phosphoethanolamine (PEA) (not yet described in commensal *Neisseria*); and (A6) development of alternative pathways to antibiotic targets (not yet described in commensal *Neisseria*). (B) Of the six described mechanisms, four have been shared from or documented to be present within *Neisseria* commensal communities and thus available for horizontal gene transfer (HGT) to pathogenic *Neisseria* through their natural competence and pilus-mediated DNA uptake machinery; the other two (A5 and A6) are yet to be described in commensal *Neisseria*. (C) Of great concern is the possibility that commensal *Neisseria* could acquire extended spectrum β-lactamase (ESBL) genes from other bacteria with which they live proximally to in the oral and nasopharynx, rendering them resistant to ceftriaxone, our last line of defense against gonorrhea. To note, chromosomal mutations encoding ceftriaxone resistance are already present in gonococcal populations (164); however, additional acquired β-lactam resistance mechanisms could hasten the wide-spread failure of this drug as an effective anti-gonococcal therapy. cN, commensal *Neisseria*; *Nci*, *N. cinerea; Ngo*, *N. gonorrhoae; Nla*, N. lactamica*; Nme*, N. meningitidis*; Nmu*, *N. mucosa; Nsu*, *N. subflava*; CAT, chloramphenicol acetyltransferase; CAMP, cationic antimicrobial peptides; SFA, sulfonamides; TMP, trimethoprim; TET, tetracycline; Chl, chloramphenicol; FDH, folate dehydrogenase; PM, polymyxins.

Full or partial gene exchange from commensals has historically played a large role in rendering antimicrobial therapies ineffective in the pathogenic *Neisseria* (see below and Fig. 1). In gonococci, reduced susceptibility to extended-spectrum cephalosporins (ESC) is predominantly associated with the acquisition of multiple commensal haplotypes at the *penA* locus, which have been reported worldwide (22, 88–91). Mosaic *penA* alleles also contribute to reduced penicillin susceptibility in gonococci and meningococci (86, 92). Furthermore, a large proportion of macrolide resistance in gonococci has been acquired from other *Neisseria* species through the transfer of mosaic *multiple transferable efflux pump* (*mtr*) alleles (22, 93–97). Mutations in the ribosomal protein genes *rplB*, *rplD*, *rpsY*, and *rpsJ* have likely been acquired from commensal *Neisseria* and correlate to azithromycin and tetracycline resistance (53, 98). Finally, meningococcal quinolone resistance has recently been reported to have been acquired through transfer of *gyrA* alleles from commensal *Neisseria* (99). These multiple documented HGT events between commensals and pathogens underscore the importance of commensals as a proximal reservoir of resistance (see Fig. 1). In Fig. 1, we also depict other well-documented mechanisms of resistance in N. gonorrhoeae that have not yet been studied in commensal *Neisseria.* These include changes of the cell surface permeability by the decoration of lipooligosaccharides (LOS) with phosphoethanolamine (PEA) (100) (Fig. 1A), and bypassing antibiotic-targeted biochemical pathways through the expression of insensitive enzymes (101) (Fig. 1A). In addition to AMR acquisition from commensal *Neisseria* (Fig. 1B), we depict the potential for ceftriaxone resistance through acquisition of novel mutations from other proximal human-associated bacterial species (Fig. 1C). Together, these examples of both intra- and intergenus AMR acquisition (discussed below) and unexplored mechanisms of resistance in commensal species highlight the importance of further characterization of commensals to fully understand the alleles at risk of rapid dissemination across species’ boundaries.

### Hybrid penicillin binding protein 2 sequences.

The first documentation of resistance transferred from commensal to pathogenic *Neisseria* was reported in 1988 by Brian G. Spratt, as part of an investigation of a gonococcal isolate collected from an individual in Boston in 1984 (86). The isolate had an elevated penicillin MIC, which prompted the comparison and discovery of a sequence with low similarity to native gonococcal alleles in the carboxy-terminal region of the transpeptidase domain of Penicillin-Binding Protein 2 (PBP2, encoded by *penA*). Shortly thereafter, the genetic basis of penicillin resistance in a N. meningitidis strain (isolated in 1978 from the United Kingdom) was also identified as being conferred by a “hybrid” (now referred to as “mosaic”) *penA* allele (92). This allele was 22% divergent from the native meningococcal sequence, containing 44 amino acid substitutions and one insertion, and was most similar to the *penA* alleles present in *N. flavescens* (now reclassified as *N. subflava*) (32). Intragenus transfer of *penA* alleles has also been reported in penicillin-resistant N. lactamica strains (52, 87, 102). In gonococci, thirty-six “flavors” or haplotypes of *penA* alleles have been described and are classified using the roman numeric system as alleles I-XXXVI, based on substitutions at 82 amino acid positions (88). Mosaic alleles in gonococci include fragments acquired from *N. cinerea* and *N. perflava* (now also *N. subflava*), or *N. perflava* (*N. subflava*) alone (5). These mutations act by both (i) lowering the affinity of the beta-lactam antibiotics (e.g., penicillin, ceftriaxone, and cefixime) for PBP2, and (ii) restricting the motions of PBP2 which are important for acylation by beta-lactams (103).

Although mosaic alleles were originally found to render gonococci and other *Neisseria* resistant to penicillin, more concerningly mosaic alleles are also correlated with decreased susceptibility to ESCs, including ceftriaxone, the last remaining option for first-line empirical antimicrobial monotherapy for gonorrhea. In a U.S. population survey from 2000 to 2013, the predominant mosaic allele (*penA* XXXIV) was found in 91% of isolates with reduced susceptibility to ceftriaxone (MIC ≥0.125 μg/mL), and 98% of isolates with reduced susceptibility to another ESC cefixime (MIC ≥0.25 μg/mL) (22). Additionally, one of the *penA* XXXIV derivatives, designated *penA41 a*nd described in the H041 isolate collected from Japan in 2009, confers high-level resistance to ceftriaxone (MIC = 2 μg/mL) (88, 104). This allele has four unique substitutions, of which the amino acid changes A311V, V316P, and T483S are key to producing high-level ceftriaxone and cefixime MICs (105). Additional mutations in mosaic alleles important for intermediate-level ESC resistance include I312M, V316T, and G545S (106, 107). A A501V substitution has also been associated with decreased ESC susceptibility (106, 108), but has only been reported in nonmosaic isolates (107). Population prevalence of mosaic alleles is alarmingly increasing in some countries. In South Korea, prevalence increased from 1.1% to 23.9% from 2012 through 2017 (89); and in Vietnam, it increased from 0% in 2011% to 27% in 2015 to 2016 (90). Therefore, though fitness costs do exist for mosaic *penA* alleles (109), sustained spread (22, 50, 89–91), repeated acquisition (22, 50), and the evolution of compensatory mutations (i.e., AcnB Q371K) in natural populations in cooccurrence (110) with these alleles points to the continued importance of commensals in transmission of clinically relevant resistance to the pathogenic *Neisseria.*

### The mosaic multiple transferable efflux pump (*mtr*) locus.

In 2012, a novel *mtrR* promoter sequence was described in a small cluster of gonococcal isolates from Australia (*n* = 10 of 397 total) with reduced susceptibility to azithromycin (≥2 μg/mL) (96). Sequences from these isolates failed to amplify on the Sequenom MassARRAY iPLEX platform, which identifies known antimicrobial resistance single nucleotide polymorphisms (SNP). Speculating mispriming due to divergent primer binding sites, the authors further characterized the locus in these isolates and found it to be more meningococcal-like than typical gonococcal promoter sequences. Since then, genomic surveys have discovered multiple “flavors” of mosaic *mtr* alleles in gonococcal populations, acquired from N. lactamica, a commensal species, and the meningococcus. Isolates with mosaic *mtr* alleles have a global distribution, and have been described in gonococci collected from the United States (22, 93), Canada (94), Australia (95, 96), and Germany (97). In a U.S. population data set, the prevalence of mosaic *mtr* sequences has increased precipitously since the recommendation to add azithromycin as part of the treatment for gonorrhea in 2012, from 1% before (2000 to 2011) to 6% (2012 to 2013) after the addition of the drug (22). Additionally, a recent 2017 New South Wales (Australia) survey estimated that 76% of reduced susceptibility to azithromycin had been derived from inheritance of mosaic *mtr* alleles (95), suggesting the persistence and expansion of these alleles in gonococcal populations over the last 2 decades globally.

The mechanistic basis of reduced azithromycin susceptibility in *mtr* mosaics stems from the interaction of multiple mutations across the *mtr* operon. The operon includes three coding domains: *mtrC* (which encodes a periplasmic membrane fusion protein), *mtrD* (encoding an inner membrane transporter of the resistance/nodulation/cell division [RND] family), and *mtrE* (encoding the outer membrane channel). The resulting tripartite efflux pump system spans the inner and outer membranes and exports diverse hydrophobic antimicrobial agents such as antibiotics, nonionic detergents, antibacterial peptides, bile salts, and gonadal steroid hormones from the cell (111–115). Using directed transformations of segments of mosaic *mtr* pump components, both Wadsworth et al. (13) and Rouquette-Loughlin et al. (15) found that two loci acting together were required to raise azithromycin MIC values above the reduced susceptibility threshold. These were: (i) a *cis*-acting single nucleotide change within the −35 A-nucleotide hexamer of the *mtrCDE* promoter which increases expression of the pump and efflux of azithromycin, and (ii) gain-of-function amino acid changes at the N- and C-terminal regions of MtrD which interacts epistatically with the promoter mutation.

Commensally acquired *mtr* sequences appear to have been acquired from N. lactamica and N. meningitidis as evidenced by high sequence similarity to these taxa (13, 15, 96, 116). Genomic signatures around the *mtr* locus were also found to be consistent with recent horizontal gene transfer and introgression of commensal alleles in the gonococcal genomic background: (i) elevated genetic diversity at loci recently acquired from other species, as these alleles would have diverged due to selection or drift in commensal strain backgrounds; (ii) increased linkage, as recombination would not have sufficient time to break down associations between loci inherited on the same recombination tract from divergent species; and (iii) admixed phylogenies between *Neisseria* species (13). These signatures have also been used to describe other loci transferred across species boundaries genome-wide (14).

### Horizontal gene transfer of ribosomal protein genes.

Antibiotics such as tetracyclines, macrolides, aminoglycosides, or chloramphenicols, target ribosomal proteins thereby reducing protein synthesis. Hence, mutations within ribosomal proteins can reduce the affinity of these antibiotics to their targets and decrease the susceptibility of the bacterium. Manoharan-Basil et al. determined whether mutations within genes encoding ribosomal proteins acquired through HGT were associated with increased azithromycin resistance in N. gonorrhoeae (98). Azithromycin, a macrolide with a bacteriostatic effect, binds to the large subunit of the ribosome (50S) and prevents the translocation of the peptidyl-tRNA, releasing incomplete and nonfunctional proteins. The 50S ribosomal subunit contains 36 ribosomal proteins. Curating the Pathogenwatch (117) and NCBI databases (including 11,644 isolates of N. gonorrhoeae and 15 commensal *Neisseria* isolates), the team identified 23 alleles out of 36 as highly conserved ribosomal genes, and performed an extensive comparative analysis to assess the likelihood of the alleles having been acquired through horizontal gene transfer (98). The authors determined that alleles of the ribosomal genes *rplB*, *rplD*, and *rplY* coding for ribosomal proteins L2, L4 and L25, respectively, were likely to have been acquired by the gonococcus from commensals through HGT. Mutations from commensal *Neisseria* within these genes were associated with MICs 4 to 16 times greater than basal susceptible azithromycin MICs of 0.125 to 0.25 μg/mL. The authors noted that these mutations, however, were not sufficient to increase MICs alone, and may be compensatory mutations impacting fitness, or epistatic, requiring additional interacting mutations to exert their effects. While the direction of acquisition of the mutations is unknown, mutations are shared mostly with *N. cinerea*, *N. mucosa*, and N. lactamica. This study concludes that HGT among *Neisseria* species is widespread in time and space. Indeed, the observations of HGT were present in the majority (46/68) of countries investigated, for isolates across time, from preantibiotic (isolates predating 1950s), golden age (between 1950 and the 1970s), and postmodern (between 1980 and the present) eras (98).

Additional resistance-encoding SNPs in loci encoding ribosomal proteins have been identified in both pathogenic and commensal *Neisseria*, suggesting their importance in different genomic backgrounds for resistance emergence, and/or as latent reserves of resistance for other species. For example, Hu et al. identified a single point mutation in the *rpsJ1* gene (previously called *tet-2*) of N. gonorrhoeae that leads to increased resistance to tetracycline. The single point mutation G → A in *rpsJ1*, leading to the amino-acid residue change V57M in the 30S ribosomal protein S10, observed in clinical isolates, appeared to increase resistance 4-fold compared with several parental laboratory strains (53). This mutation was also observed in commensal *Neisseria* species (118). However, a definitive correlation between tetracycline resistance and the V57M substitution was not observed, suggesting that additional mutations in *mtrR* and *penB* may also be needed to develop high levels of resistance to tetracycline (53). Although this example does not directly identify HGT of this particular SNP between commensal and pathogenic *Neisseria*, it highlights conserved mechanisms of resistance broadly distributed across the genus and suggests their immediate availability for rapid resistance transfer.

### Transfer of quinolone resistance to N. meningitidis from commensals.

Reports of ciprofloxacin resistance in meningococcal populations are increasing and have been documented globally (119–121). In part, these cases are due to HGT from the commensal *Neisseria.* The first records of commensally acquired quinolone resistance in meningococci are from the United States in 2007 and 2008. Three cases of meningococci from serogroup B were identified as ciprofloxacin-resistant (with MICs of 0.25 μg/mL) in two states (North Dakota and Minnesota). These isolates all had acquired a T91I substitution in the gene encoding DNA Gyrase Subunit A (*gyrA*) from N. lactamica, which rendered them resistant. A more recent survey in China found a high prevalence of quinolone resistant meningococci (>70%) with multiple divergent *gyrA* and *parC* (encoding topoisomerase IV) haplotypes (99). Isolates with elevated MICs (≥0.25 μg/mL) had all acquired the same T91I mutation as the North American isolates mentioned above, with 53.4% of resistant alleles being acquired from the commensals: N. lactamica, *N. cinerea*, and *N. subflava.* Chen et al. also found that of 293 commensal *Neisseria* sampled, 99% were quinolone resistant, suggesting that quinolone resistance may have an especially high likelihood of interspecies transfer from commensal reservoirs to pathogens (99).

## ANTIBIOTIC SELECTION ON COMMENSALS: FREQUENT BYSTANDERS TAKE UP ARMS

Antibiotic resistance in many bacterial species increases with the consumption of antibiotics in the community (122–126), and in gonococci several studies have suggested a link between antibiotic use and the emergence and spread of AMR (29, 30). For example, a survey across 24 European countries found that cephalosporin and fluoroquinolone use significantly increased ceftriaxone/cefixime and ciprofloxacin MICs, respectively (127). Increased azithromycin MICs have also been reported after azithromycin usage in patients at a Dutch sexual health clinic (128) and at a U.S. clinic in Portland (129). Additionally, seasonal variability in azithromycin use in the United States (increased in the winter compared with other months), has been correlated with seasonal fluctuations in azithromycin MICs in N. gonorrhoeae populations (higher in the winter and spring compared with summer and fall [130]). Finally, more generally, resistance has emerged to all new antimicrobials used to treat gonorrhea in only a few years or decades after their initial recommendation (131). These examples are unsurprising, and simply a function of the selective pressure of antibiotic use killing off susceptible bacteria, followed by the survivors (the more resistant members of those populations) propagating in ecological niches that become vacant postantibiotic treatment.

Importantly, most of the aforementioned studies investigating a link between antibiotic use and resistance support bystander selection as a main driver in the evolution of AMR. Bystander selection is the unintentional selection of resistance in bacteria not targeted by the prescribed antimicrobial therapy, and is higher in bacteria with high carriage rates, as they more frequently experience exposure to consumed drugs (132). Extrapolating this finding to *Neisseria* suggests that commensals (carriage rate ~100%) should experience higher bystander selection from antibiotic use compared with their pathogenic relatives (carriage rate between 0.01 and 10%) (29, 30, 133), and should be more likely to “take up arms” and display resistance to clinically important antimicrobials. Reduced drug susceptibility in commensal *Neisseria* populations has been shown to be directly selected for after antibiotic usage in a study of Vietnamese men, in which recent antibiotic use (within 1 month) was strongly associated with increased MICs to cefixime, ceftriaxone, and cefpodoxime in commensal *Neisseria* compared with a control population with no antibiotic use between 1 and 6 months prior to the study (28). This work highlights the impact of bystander selection on commensals, their persistent threat as evolutionary engines for resistance donation, and the underlying importance of characterizing the resistance phenotypes and genotypes of commensals to fully describe the *Neisseria* resistome.

## THE CURRENT STATE OF WGS SURVEILLANCE IN COMMENSAL *NEISSERIA*

Currently, the characterization of resistance in commensal reservoirs has only been conducted by individual academic laboratory groups that have often focused on a small sample of strains, rather than a concerted effort, nationally supported, on a large population of clinical or general population isolates (27, 99, 134–136). Hence, there is a disproportionate amount of genomic data for N. gonorrhoeae and N. meningitidis isolates, compared with the number of deposits for commensal *Neisseria* species. A search of WGS deposits to the SRA (June 2022) revealed that pathogenic species, N. gonorrhoeae and N. meningitidis, are 100 to 1,000 times more represented compared with commensal species (N. gonorrhoeae: 42,799, N. meningitidis: 34,050, N. lactamica: 1,166, and less than 100 for each of the other human commensal *Neisseria* species, as of June 21, 2022). This undoubtedly leads to a molecular bias in comparative genomics analyses impacting findings and conclusions on *Neisseria* historic and contemporary evolution, including interspecific AMR gene transfer events (e.g., their frequency, presence, direction, etc.). A broad WGS database associated with AMR profiles of commensal *Neisseria* species is necessary for the scientific community to further anticipate the evolution of AMR in pathogenic species. We are at a critical inflection point for the maintenance of effective antimicrobial treatment of N. gonorrhoeae, and only with a better understanding of the spread of AMR across *Neisseria* species will we be able to develop better antimicrobial stewardship strategies against gonorrhea.

Most available studies linking MIC data to genomic sequences focus on small panels of commensals that are publicly available, or isolated by independent research groups. For example, Fiore et al. described a panel of 26 *Neisseria*, of which 14 isolates are typically considered human commensals, which are publicly available through the CDC and Food and Drug Administration’s (FDA) Antibiotic Resistance (AR) Isolate Bank within the “*Neisseria* species MALDI-TOF Verification panel” (118). Of these isolates, 10 had reduced susceptibility to at least one of the antibiotics tested (penicillin, ceftriaxone, cefixime, tetracycline, azithromycin, or ciprofloxacin); and the single *N. bacilliformis* was resistant to all tested drugs. Notably, all the *N. subflava* isolates (*n* = 5) were resistant to azithromycin, and some also displayed reduced susceptibility to tetracycline, penicillin, and cefixime. The authors coupled MIC test results with genome sequence to identify putative resistance-encoding mutations and found several known loci or alleles previously described in gonococci, including: *gyrA* (S91F), *tetM*, and TEM-type β-lactamases. The authors also noted that not all resistance-contributing mutations were able to be described in the study, evidenced by isolates with the same resistance haplotypes displaying variation in MIC. For example, two *N. subflava* isolates had MICs to penicillin of 3 and 1 μg/mL, despite having the same mutations in *penA* and *porB.* The field will remain severely limited in the genetic diversity of commensals represented given the extreme scarcity of commensal samples stored at public strain repositories; this highlights the importance of increasing the support for commensal collection through additional mechanisms (described below).

A thorough and comprehensive evaluation of the availability of published commensal genomic sequences with associated antimicrobial resistance data has recently been conducted by Vanbaelen et al. (30). In their systematic review, the authors found that despite over 295 published studies on commensals, only 17 contained sufficient data to link WGS with MIC values. Reasons for exclusion of the remaining studies included: No MIC data (*n* = 179), only a single isolate reported (*n* = 9), MIC data present but for nonrelevant antimicrobials (*n* = 6), animal-associated *Neisseria* reported (*n* = 3), missing data (*n* = 84), repeated characterization of an isolate (*n* = 1), and inferred MIC values (*n* = 1). Of the 15 retained studies (two studies were not accessible), the number of isolates characterized ranged from 4 to 491, with a mean of 115.3 ± 31.82 isolates per study. However, those collecting larger numbers of isolates within this list contained N. gonorrhoeae and N. meningitidis isolates as the primary focal species sampled. Limiting the list to those studies that only focused on collection of commensals (*n* = 10) the upper limit of isolates drops from 4 to 286, and the mean shrinks to 88 ± 30.0 isolates per study. Despite the small number of studies identified, key epidemiological and biological information was obtained such as: (i) commensal species do not appear to be intrinsically resistant, (ii) commensal species have increasing MIC values over time in multiple countries, and (iii) AMR prevalence in commensal species is a clear threat to AMR in pathogenic *Neisseria.* The paucity of commensal data sets, and their limited and biased geographic sampling (11 from Europe, five from Asia, one from the United States), identified by this comprehensive analysis of available data strongly supports the need for increased characterization and sequencing of commensal species through a broader national or international strategy. As we propose below, we believe that these data could be generated through established surveillance programs, and this expansion could provide the necessary pool of commensal *Neisseria* for a broad sampling of diversity within the genus, for the analysis of AMR profiles and sequence correlation to AMR.

## CANARY IN THE COAL MINE: THE BENEFITS OF SURVEILLANCE IN COMMENSALS

**FIG 2:**
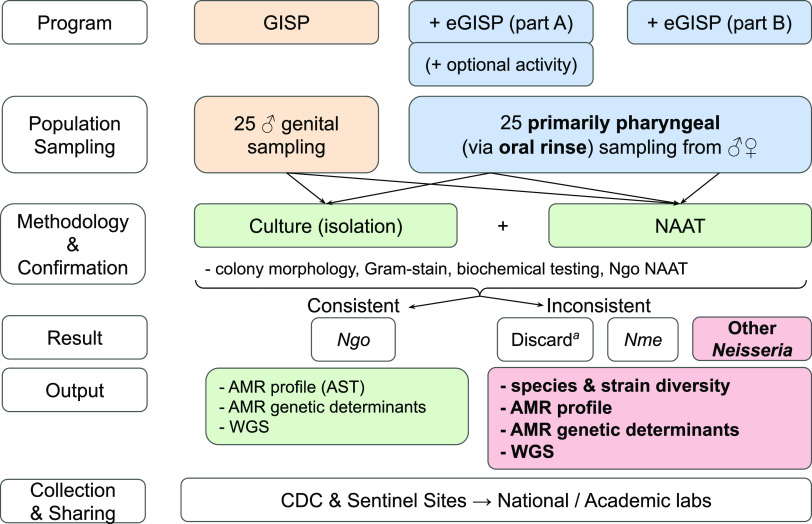
Flowchart of the current protocol for the Gonococcal Isolate Surveillance Project (GISP, run by the CDC). For each clinical collection site, the current GISP (orange) and eGISP (blue) programs sample 25 individuals from genital and 25 from nongenital sites per month, respectively. Currently, all samples are submitted for morphological testing and nucleic acid amplification testing (NAAT) and retained if bacterial isolates are Gram-negative, diplococci, oxidase-positive, have a tan/transparent colony morphology on modified Thayer-Martin media, and NAAT-positive for N. gonorrhoeae (Ngo)*. ^a^*Sentinel sites not participating in eGISP discard their non-Ngo samples. Retained colonies are then subjected to AMR profiling and WGS. We propose a minor modification to the eGISP program (bold and red), in which all pharyngeal samples collected via oral rinse-and-gargle (138) are conserved if they display features inconsistent with N. gonorrhoeae, yet consistent with other members of the *Neisseria* genus (Gram−, diplococci or rods, and oxidase+) (bold and red). We also suggest that pharyngeal samples are inoculated onto LBVT.SNR media, which is selective for commensal *Neisseria.* Subsequent development of a pipeline for complete analysis of each resultant non-N. gonorrhoeae strain (AMR profiling, WGS, etc.) will allow for detailed characterization of *Neisseria* species and strain diversity, allowing for a comprehensive evaluation of the *Neisseria* resistome and other comparative genomics analyses of interest.

The importance of commensals as a reservoir for resistance donation has led Kenyon et al. to propose a “pan-*Neisseria”* approach to selecting drugs for treatment of pathogenic *Neisseria* infections which should limit the selection of resistant commensal species, and prevent the spread of resistance determinants to pathogenic species (27). Building upon those goals, we call for the expansion of collection protocols to include commensal *Neisseria* to aid in the fight against AMR in pathogenic *Neisseria.* Specifically, we recommend that a point be added to the eGISP-optional analysis section (137), so that the collection of *Neisseria* from extragenital sites, from male and female patients with possible N. gonorrhoeae exposure (*n* = 25 per sentinel site per month), includes additional human-borne *Neisseria* species and is not restricted to pathogenic *Neisseria*. (Fig. 2). To achieve this, we recommend prioritizing oropharyngeal samples, which are more likely to harbor commensals than rectal samples, through oral rinse-and-gargle to collect diverse *Neisseria* species from distinct ecological sites in the mouth and nasopharynx, as described by Laumen et al. (138). The oral rinse-and-gargle method has been demonstrated to capture increased *Neisseria* species diversity as opposed to a posterior oropharyngeal/tonsillar swab, is less invasive than a swab, and is more readily implementable as a standardized tool across clinics (138).

In addition, the current eGISP protocol isolates pathogenic *Neisseria* from patient samples through the use of a selective medium (modified Thayer-Martin media [MTM]) containing vancomycin (inhibits Gram-positive bacteria), nystatin (inhibits fungi), and colistin and trimethoprim (which inhibit many Gram-negative bacteria, but not pathogenic *Neisseria*) (137). However, commensal *Neisseria* display variable sensitivity to colistin which limits the utility of MTM media for sampling commensals (139, 140). Therefore, we suggest the use of an additional plate of complementary medium, specifically designed to isolate commensal *Neisseria* (LB containing vancomycin, trimethoprim, sucrose, neutral red, LBVT.SNR) (19, 138). While labor-intensive, this new strategy will undoubtedly create a wider and more diverse collection of commensal *Neisseria* than is currently available. Downstream morphology-, biochemical-, and genomic-based approaches (currently undertaken as part of eGSIP) should be used to further characterize the commensal *Neisseria* species. The integration of genus-wide *Neisseria* NAATs (e.g., sequencing of *rplF* is sufficient to differentiate pathogenic and commensal *Neisseria* species) will further aid in selecting commensal *Neisseria* for downstream analytical pipelines (141).

Once isolates are obtained, AMR profiling and WGS bioinformatics workflows used with N. gonorrhoeae can be expanded to newly isolated commensal *Neisseria* (142, 143). Alternatively, low-cost semiquantitative AMR profiling, by adding antibiotics of interest at an established concentration to plates, could be used to further select for AMR *Neisseria* (138). WGS and AMR profiling data can be used as the input for established bioinformatics pipelines. For example, the combined PubMLST (https://pubmlst.org/neisseria) and Bacterial Isolate Genome Sequence Database (BIGSdb) platforms (144, 145), which store both (i) information on gene-by-gene allelic variation across genera and species, and (ii) an isolate database with associated metadata and sequencing data, already have workflows for identification of AMR loci across *Neisseria* spp. The utility of these platforms has recently been demonstrated in describing associations between AMR, the presence of accessory genes, and population structure within N. gonorrhoeae (146). We believe that expanding this type of approach to commensal species could enhance epidemiological surveillance and alert us to early warning indicators of new resistance phenotypes and genotypes that could sweep through pathogenic *Neisseria* populations and possibly cause resistance outbreaks.

Therefore, the surveillance of commensal *Neisseria* species would require minor changes in the methodology of the clinical microbiology lab, and would not modify patients’ experiences nor sample collection at large (138). We acknowledge that our proposed modification to eGISP (adding the collection, characterization, and storage of commensal isolates) will be both a costly endeavor and require significantly increased effort for the personnel involved in surveillance programs; however, we also believe that our current blindness of known AMR reservoirs for *Neisseria* pathogens is a key gap in addressing AMR within the genus. Implementing this approach as a pilot within a single program (e.g., GISP) for a limited time, may thus prove a necessary first step to assess the overall impact of scaling modified eGISP to worldwide surveillance efforts (e.g., GASP and Euro-GASP). Ultimately, this expansion could uncover the global diversity of *Neisseria* species, the AMR phenotypes and genotypes harbored, and the prevalence of allelic exchange across species’ boundaries (see 14).

Complementary lab-based approaches to broad field-work surveillance of AMR in *Neisseria* include transformation of susceptible strains of different species with DNA from a resistant donor strain, and experimental evolution. Transformation experiments have proven useful and effective in uncovering molecular mechanisms of antimicrobial resistance (5, 61, 147). These experiments can be conducted intraspecies, and interspecies but with lower efficiency across species due to the barriers described previously, and the likely dependence of AMR expression on additive or epistatic interactions in divergent genomic backgrounds. Additionally, *in vitro* evolution experiments can reveal the spontaneous mutations caused by DNA replication and repair errors which increase mean fitness in new selective environments (148), and are quick and easy to implement in the research laboratory due to the short generation times of *Neisseria* (~60 min). Using this approach, multiple tandem duplications in the locus encoding the 50S ribosomal L34 protein (*rpmH*) and the intergenic region proximal to the 30S ribosomal S3 protein (*rpsC*) were identified to increase resistance (≥2 μg/mL) to the macrolide antibiotic azithromycin in the commensal *N. elongata* in 20 days (or approximately 480 generations) (149). A similar experiment also described an identical 7LKRTYQ12 sequence duplication in *rpmH* as a transient steppingstone to high level azithromycin resistance in N. gonorrhoeae (150). Multiple mutations, in particular in *penA* (encoding a A501V substitution) and *ftsX* (encoding a T31P substitution), have also been uncovered as causal to reduced ceftriaxone susceptibility in N. gonorrhoeae through *in vitro* selection (108). Thus, expansion of these experiments to additional drug and species combinations could help characterize the potential *Neisseria* resistome, improving genotype-based surveillance efforts (WGS or probe-based) which depend on a known database of resistance determinants in both pathogenic and commensal species within the genus. A key caveat of such evolutionary approaches is that mutations evolving *in vitro* may not be representative of those that can persist in natural populations. Despite this limitation, lab-based evolution experiments can still provide valuable information on potential resistance genes and should be viewed as complementary to the expansion of the surveillance program mentioned above.

One additional concern highlighting the immense importance of global surveillance across the *Neisseria* is the growing prevalence of transferable plasmids containing extended spectrum β-lactamase (ESBL) genes, which are present in ~30 human-associated bacterial species (151). ESBLs confer resistance to broad-spectrum cephalosporins with oxyimino side chains (e.g., ceftriaxone) and other β-lactam antibiotics (152), and were designated a serious and increasing threat within the CDC’s 2019 Antibiotic Resistance Threat Report (153). As commensal *Neisseria* species coevolve in the nasopharynx with respiratory tract pathogens which harbor plasmids containing ESBL genes (Fig. 1C), there is a serious threat that a commensal *Neisseria* could acquire these ESBL loci rendering them resistant to ceftriaxone, our last line of defense against gonorrhea. From there, either full or partial transfer of ESBL encoding sequences from carrying commensal *Neisseria* to N. gonorrhoeae may lead to a disastrous outcome for gonococcal treatment. Indeed, acquisition of β-lactamase expressing plasmids in the 1970s by N. gonorrhoeae from H. influenzae (154), which have persisted in contemporary gonococcal populations (22), imparted resistance to penicillin (typically > 8 μg/mL), and was a main driver for the discontinued use of penicillin as a treatment for gonorrhea. Considering the frequent genetic exchange across *Neisseria* species (11, 14, 23), known examples of gene transfer from other bacteria to *Neisseria* (154–156), and broad mobilization and dissemination of ESBL-containing plasmids across bacteria species and genera (157–159), we must consider the above nightmare scenario which underscores the importance of surveillance of commensals, coresidents of the same ecological niche as ESBL-carrying respiratory tract pathogens.

In closing, we highly encourage the *Neisseria* research community to support and focus on increasing the collection of commensal *Neisseria* by both clinical and academic laboratory groups, and sequence these isolates to broaden the database of genomic data for commensal *Neisseria* species. Not only will these isolates and corresponding sequence data sets aid in the fight against resistance by characterizing *Neisseria* alleles available for intragenus transfer, they may also aid the *Neisseria* research community by providing a pool of data to study other common/divergent genomic features and *Neisseria* biology at large, such as the presence and importance of prophages (160), the diversity of anti-phage defense systems (161), the identification of toxin/antitoxin systems (146), patterns of killing through DNA methylation (83), and identifying immunogenic antigens as candidate vaccines (162).

## CONCLUSION

AMR in commensal *Neisseria* species serves as a reservoir for AMR in pathogenic *Neisseria* species, in particular N. gonorrhoeae. N. gonorrhoeae has evolved resistance mechanisms which reduce susceptibility to all antimicrobials used as first-line therapies for the treatment of uncomplicated gonorrhea, and as this review demonstrates, they are often acquired through horizontal gene transfer from closely related commensals. Commensal *Neisseria*, which are carried in nearly 100% of the human population, undergo unintentional antimicrobial treatment and selection, making these “bystanders” major participants in the acquisition, maintenance, and transfer of AMR determinants. Surveillance of gonorrhea is critical for sexually transmitted infection prevention and addressing AMR (163). As such, we propose that expanding surveillance to all *Neisseria* species will greatly strengthen our capacity to monitor AMR in N. gonorrhoeae and predict and react to antimicrobial resistance outbreaks across the globe by locating early warning indicators (i.e., AMR determinants) that could be on the verge of rapid dissemination into pathogenic *Neisseria* populations. Finally, surveillance, isolation, collection, sequencing, and analysis of commensal *Neisseria* genomes could broadly expand our understanding of the biology and AMR determinants within the *Neisseria* genus at large, and may provide novel insights into new strategies to treat and prevent pathogenic *Neisseria* infections. Ultimately, commensal *Neisseria* are key drivers of AMR emergence in important human pathogens, and thus it is critical for the *Neisseria* research community to consider supporting, developing, and funding the timely integrations of commensal *Neisseria* into established gonococcal clinical surveillance programs worldwide. We believe that public access to the resultant omics and phenotypic data sets will be critical to generating new insights for addressing AMR in the pathogenic *Neisseria*, species of global concern to public health.
